# Evolving Paradigms in Minimal Access Surgery: A Comprehensive Review of Single-Incision Laparoscopic Appendicectomy

**DOI:** 10.7759/cureus.102013

**Published:** 2026-01-21

**Authors:** Divyakant H Barot, Minesh Sindhal, Priyanka Aanandaka, Nidhi D Gheewala, Parmar Bhargav

**Affiliations:** 1 General Surgery, All India Institute of Medical Sciences, Rajkot, Rajkot, IND; 2 Surgery, All India Institute of Medical Sciences, Rajkot, Rajkot, IND

**Keywords:** appendectomy, general surgery, healthcare tech, minimal access surgery, sila (single incision laparoscopic appendectomy), sils (single incision laparoscopic surgery), single incision laparoscopic appendectomy, single-port, surgical-glove port

## Abstract

Single-incision laparoscopic appendicectomy (SILA) has emerged as an evolution of minimally invasive surgery, aiming to minimize access trauma while maintaining the advantages of conventional laparoscopic appendicectomy (CLA). This review analyzes the cumulative evidence from 24 PubMed-indexed studies comparing SILA and CLA, with a focus on safety, operative outcomes, cosmesis, and the cost-saving impact of glove-port innovations.

A structured literature review was performed using PubMed, including 24 studies (12 randomized controlled trials, seven meta-analyses, three retrospective cohorts, and two feasibility studies). Data were extracted for operative duration, pain scores, complications, hospital stay, cosmetic satisfaction, and economic parameters. Studies involving pediatric, adult, and complicated appendicitis cases were included.

Across 10,968 patients (SILA = 5,246; CLA = 5,722), no significant difference was observed in major complications, hospital stay, or infection rates. SILA demonstrated shorter recovery (3.8 vs. 4.5 days), lower pain scores (Visual Analogue Scale (VAS) 3.2 vs. 3.5), and superior cosmetic satisfaction (9.1 vs. 7.8). The mean operative time was initially longer for SILA (49.8 vs. 42.5 minutes) but equalized after 20-25 cases. Glove-port SILA reduced costs by 45-60% compared to CLA. The overall wound infection rate was 2.5% for SILA versus 2.7% for CLA, and umbilical hernia occurrence was 1.3% versus 0.9%, respectively.

SILA offers comparable safety and faster recovery than CLA, with enhanced cosmetic outcomes and substantial cost benefits using glove-port techniques. Once the learning curve is surpassed, SILA is an effective, safe, and accessible alternative for uncomplicated appendicitis, especially in cost-conscious healthcare settings.

## Introduction and background

Appendicitis remains one of the most common general surgical emergencies globally. Since its first laparoscopic application in the 1980s, laparoscopic appendicectomy has become the standard of care for uncomplicated appendicitis, offering reduced postoperative pain, fewer wound complications, and shorter recovery times compared to open surgery [1].

As technology and surgical skills advanced, attention shifted toward reducing the number and size of ports to minimize surgical trauma. This gave rise to single-incision laparoscopic surgery (SILS), a concept rooted in natural orifice transluminal endoscopic surgery (NOTES) principles [2].

Single-incision laparoscopic appendicectomy (SILA) performs the entire procedure through a single umbilical incision, concealing the scar and potentially reducing infection risk and postoperative pain [3]. Initially performed with costly commercial single-port systems, SILA has evolved through cost-effective modifications such as the glove-port, which uses standard surgical instruments and disposable materials [4].

This review aims to synthesize current evidence on SILA, examining its evolution, outcomes, technical aspects, learning curve, and cost-effectiveness, with an emphasis on the glove-port approach as an affordable and practical innovation.

## Review

Evolution of minimally invasive appendicectomy

Traditional multi-port laparoscopic appendicectomy (CLA) employs three ports and remains a benchmark procedure in general surgery. SILA emerged to minimize access trauma while preserving laparoscopic benefits [5]. Early reports in the late 2000s demonstrated the feasibility of SILA but concurrently highlighted technical and ergonomic challenges, including reduced triangulation and instrument crowding [6]. Over time, these limitations have been mitigated by refinements in instrumentation (articulating instruments and angled optics) and improved surgeon experience, which together have reduced the initial technical difficulty and broadened the indications for SILA [7].

Surgical technique and the glove-port innovation

**Figure 1:**
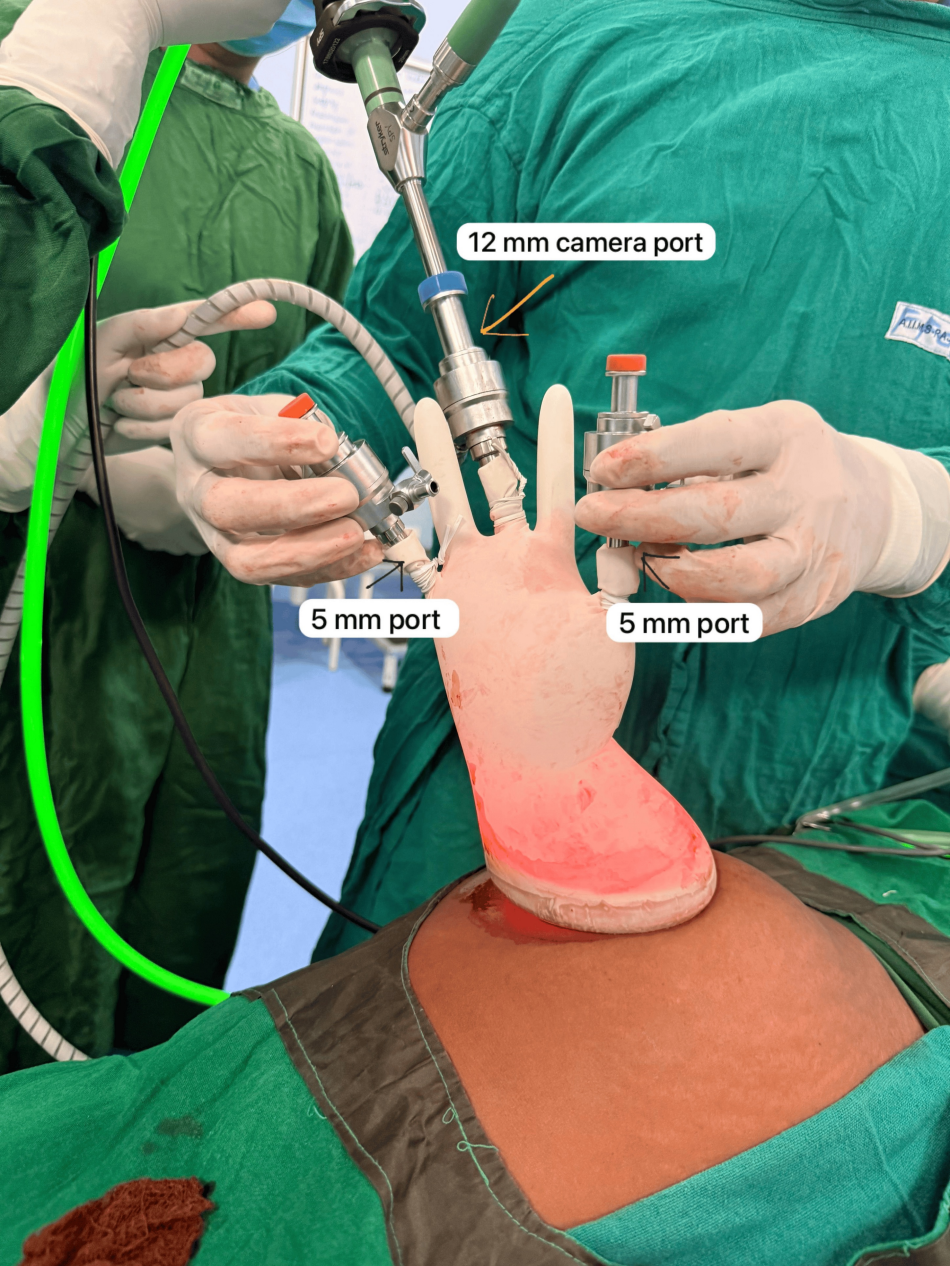
intraoperative view of glove-port set up for single-incision laparoscopic appendicectomy (SILA) Image reproduced with permission from the author’s institutional collection, All India Institute of Medical Sciences, Rajkot, India.

In SILA, a 2-2.5 cm horizontal infra-umbilical incision is made, and a wound retractor is inserted. A sterile glove is fixed to the outer ring, with trocars inserted into the glove fingers. Pneumoperitoneum is maintained at 12-14 mmHg. The glove-port configuration (Figure 1) permits the entire appendicectomy to be performed through a single infra-umbilical incision while preserving adequate visualization and instrument mobility. After insertion of the wound retractor and creation of the glove-port, the appendix is identified; the mesoappendix is divided using monopolar or bipolar cautery according to surgeon preference, and the appendiceal base is secured with an endoloop or an intracorporeal knot prior to division [8]. Specimen retrieval is then accomplished through the glove-port sleeve, avoiding additional skin incisions and minimizing manipulation of the wound edges. This technique therefore maintains the procedural steps of conventional laparoscopy while improving cosmesis and reducing additional port-related trauma. This method drastically reduces cost compared with commercial single-port devices while maintaining excellent visibility and range of motion [9].

Comparative outcomes

Meta-analyses have consistently shown no significant difference in complication rates between SILA and CLA [10,11], reinforcing that the newer single-incision approach offers equivalent safety and postoperative outcomes. These findings support its role as a viable alternative for uncomplicated appendicitis, especially in centers familiar with laparoscopic protocols.

Key comparative findings between SILA and CLA have been summarized from several comparative and randomized studies [12-16]. These investigations consistently demonstrate comparable safety, similar hospital stay durations, and nearly equivalent postoperative pain scores between the two approaches. However, SILA generally offers superior cosmetic satisfaction and a modest reduction in analgesic requirements. The differences in operative time are largely attributable to the learning curve and gradually diminish after approximately 20-25 cases. Table 1 compiles the principal outcome measures from these representative studies to provide a consolidated overview of clinical performance between the two techniques. Studies were included if they involved pediatric or adult patients with uncomplicated acute appendicitis undergoing single-incision laparoscopic appendicectomy. Eligible study designs included randomized controlled trials, meta-analyses, retrospective cohort studies, and feasibility studies. Exclusion criteria comprised perforated or gangrenous appendicitis, diffuse peritonitis, pregnancy, morbid obesity, and prior lower abdominal surgery.

**Table 1:** Comparison between single-incision laparoscopic appendicectomy (SILA) and conventional laparoscopic appendicectomy (CLA). Data compiled from multiple published studies [12-16], reproduced with author permission under educational use license.

Parameter	Single-Incision Laparoscopic Appendicectomy (SILA)	Conventional Laparoscopic Appendicectomy (CLA)
Number of Ports	Single umbilical incision (glove-port or commercial port)	Three ports (umbilical, suprapubic, left lower quadrant)
Operative Time	Initially longer (45–65 min), comparable after 20–25 cases	30–50 min
Postoperative Pain	Equal or slightly lower within 24 h	Comparable
Hospital Stay	1–2 days (no difference)	1–2 days
Wound Complications	0–4%	0–5%
Umbilical Hernia	0–2% (if fascia closed properly)	<1%
Cosmetic Outcome	Superior (hidden umbilical scar)	Moderate (visible scars)
Analgesic Requirement	Slightly reduced	Standard requirement
Return to Activity	3–5 days	4–6 days
Cost	Low with glove-port; high with commercial ports	Moderate
Learning Curve	15–25 cases for proficiency	Short; widely established
Feasibility in Complicated Appendicitis	Limited, improving with experience	Established safety
Overall Safety	Comparable to CLA	Proven standard
Patient Satisfaction	Higher due to better cosmesis	High but lower aesthetics

Learning curve and ergonomics

The learning curve for SILA typically stabilizes after 20-25 procedures, during which the surgeon gains proficiency in parallel instrument movement and develops an intuitive understanding of the single-axis visual field [17]. Early in the learning phase, reduced triangulation and instrument crowding present ergonomic challenges that can prolong operative duration and increase surgeon fatigue. However, structured training programs incorporating simulation-based practice, hands-on workshops, and proctored mentorship have been shown to significantly shorten the learning period and improve procedural confidence [18].

Adaptation to SILA requires deliberate ergonomic adjustments compared to three-port CLA. The surgeon and camera assistant must align coaxially, maintaining consistent camera stability and avoiding instrument crossing. The use of flexible or articulating instruments, 30° laparoscopes, and angled trocars can restore triangulation and facilitate smoother dissection within the limited workspace [19]. These ergonomic refinements improve surgeon comfort and precision, particularly in cases involving a retrocecal appendix or dense mesoappendix.

Although the learning curve is steeper than for conventional laparoscopy, studies demonstrate that once competence is achieved, operative time, intraoperative complication rate, and conversion to multi-port techniques do not significantly differ from CLA. Moreover, institutions integrating SILA into their residency curricula have reported improved procedural efficiency after 15-20 supervised cases, reinforcing that with adequate exposure, SILA is a reproducible and safe technique even for early-career surgeons.

Economic feasibility

Economic feasibility is a major determinant of whether SILA can be adopted widely, particularly in low- and middle-income countries. The initial limitation to SILA’s global acceptance was the high cost of commercial single-port platforms, which could exceed several times the expense of standard three-port laparoscopic setups. This economic barrier prompted the development of cost-effective alternatives such as the glove-port system - constructed from a wound retractor and a sterile surgical glove - allowing surgeons to perform SILA with standard instruments at a fraction of the cost [20].

Multiple comparative studies have demonstrated that glove-port SILA can reduce material expenditure by 45-90% without compromising patient safety or operative outcomes [20,21]. The disposable glove and reusable wound retractor are inexpensive, readily available, and easily assembled, making the technique especially advantageous for government and teaching hospitals. In addition to equipment savings, reduced postoperative analgesic requirements, shorter recovery periods, and earlier discharge contribute further to the overall cost-effectiveness of SILA compared with CLA.

From a health systems perspective, the glove-port approach exemplifies appropriate technology for resource-limited settings. By minimizing dependence on proprietary access systems and optimizing the use of existing instruments, SILA democratizes minimal access surgery in district-level facilities. As experience accumulates and learning curves flatten, the balance of cost, safety, and efficiency positions SILA - especially in its glove-port adaptation - as a sustainable, patient-centered evolution of minimally invasive appendicectomy.

Future directions

The future of SILA lies in robotic integration, 3D visualization, and AI-assisted intraoperative guidance [21]. Artificial intelligence is expected to assist in video analytics, skill training, and outcome prediction [22]. Ongoing multicenter trials are expanding SILA indications to complicated appendicitis, obesity, and pediatric cases, aiming to establish universal procedural guidelines [23,24].

## Conclusions

SILA exemplifies the advancement of minimal access surgery. The technique is safe, reproducible, and cosmetically superior to multi-port laparoscopy. The glove-port adaptation has democratized its use, particularly in resource-limited regions.

With further refinement, structured training, and robust long-term data, SILA is poised to become a standard alternative to CLA worldwide.

## References

[1] Schildberg C, Weber U, König V (2025). Laparoscopic appendectomy as the gold standard: what role remains for open surgery, conversion, and disease severity?: An analysis of 32,000 cases with appendicitis in Germany. World J Emerg Surg.

[2] Greaves N, Nicholson J (2011). Single incision laparoscopic surgery in general surgery: a review. Ann R Coll Surg Engl.

[3] Han Y, Yuan H, Li S, Wang WF (2024). Single-incision versus conventional three-port laparoscopic appendectomy for acute appendicitis: a meta-analysis of randomized controlled trials. Asian J Surg.

[4] Carrillo Montenegro AF, Aristizabal Rojas S, Pulido Segura JA (2023). Single incision laparoscopic appendectomy with surgical-glove port is cost-effective and reliable in complicated acute appendicitis: a casecontrol multicenter study in Colombia. Heliyon.

[5] St Peter SD, Adibe OO, Juang D (2011). Single incision versus standard 3-port laparoscopic appendectomy: a prospective randomized trial. Ann Surg.

[6] Xue C, Lin B, Huang Z, Chen Z (2015). Single-incision laparoscopic appendectomy versus conventional 3-port laparoscopic appendectomy for appendicitis: an updated meta-analysis of randomized controlled trials. Surg Today.

[7] Aly OE, Black DH, Rehman H, Ahmed I (2016). Single incision laparoscopic appendicectomy versus conventional three-port laparoscopic appendicectomy: a systematic review and meta-analysis. Int J Surg.

[8] Dapri G, Casali L, Dumont H (2011). Single-access transumbilical laparoscopic appendectomy and cholecystectomy using new curved reusable instruments: a pilot feasibility study. Surg Endosc.

[9] Liang HH, Hung CS, Wang W (2014). Single-incision versus conventional laparoscopic appendectomy in 688 patients: a retrospective comparative analysis. Can J Surg.

[10] Cai YL, Xiong XZ, Wu SJ (2013). Single-incision laparoscopic appendectomy vs conventional laparoscopic appendectomy: systematic review and meta-analysis. World J Gastroenterol.

[11] Irfan A, Rao A, Ahmed I (2025). Single-incision versus conventional multi-incision laparoscopic appendicectomy for suspected uncomplicated appendicitis. Cochrane Database Syst Rev.

[12] Carter JT, Kaplan JA, Nguyen JN, Lin MY, Rogers SJ, Harris HW (2014). A prospective, randomized controlled trial of single-incision laparoscopic vs conventional 3-port laparoscopic appendectomy for treatment of acute appendicitis. J Am Coll Surg.

[13] Frutos MD, Abrisqueta J, Lujan J, Abellan I, Parrilla P (2013). Randomized prospective study to compare laparoscopic appendectomy versus umbilical single-incision appendectomy. Ann Surg.

[14] Irfan A, Rao A, Ahmed I (2024). Single-incision versus conventional multi-incision laparoscopic appendicectomy for suspected uncomplicated appendicitis. Cochrane Database Syst Rev.

[15] Kim WJ, Jin HY, Lee H (2021). Comparing the postoperative outcomes of single-incision laparoscopic appendectomy and three port appendectomy with enhanced recovery after surgery protocol for acute appendicitis: a propensity score matching analysis. Ann Coloproctol.

[16] Buckley FP 3rd, Vassaur H, Monsivais S, Jupiter D, Watson R, Eckford J (2014). Single-incision laparoscopic appendectomy versus traditional three-port laparoscopic appendectomy: an analysis of outcomes at a single institution. Surg Endosc.

[17] Lee GR, Kim JH, Kim CH, Lee YS, Kim JJ (2021). Single-incision laparoscopic appendectomy is a safe procedure for beginners to perform: experience from 1948 cases. Surg Endosc.

[18] Gates NL, Rampp RD, Koontz CC, Holcombe JM, Bhattacharya SD (2019). Single-incision laparoscopic appendectomy in children and conversion to multiport appendectomy. J Surg Res.

[19] Wu S, Shen Y, Wang J, Wei J, Chen X (2022). Conventional three-port laparoscopic appendectomy versus transumbilical and suprapubic single-incision laparoscopic appendectomy using only conventional laparoscopic instruments. Langenbecks Arch Surg.

[20] AlHabeeb W (2022). Heart failure disease management program: A review. Medicine (Baltimore).

[21] Kye BH, Lee J, Kim W, Kim D, Lee D (2013). Comparative study between single-incision and three-port laparoscopic appendectomy: a prospective randomized trial. J Laparoendosc Adv Surg Tech A.

[22] King A, Fowler GE, Macefield RC (2025). Use of artificial intelligence in the analysis of digital videos of invasive surgical procedures: scoping review. BJS Open.

[23] Zavras N, Vaos G (2020). Management of complicated acute appendicitis in children: still an existing controversy. World J Gastrointest Surg.

[24] Pan Z, Jiang XH, Zhou JH, Ji ZL (2013). Transumbilical single-incision laparoscopic appendectomy using conventional instruments: the single working channel technique. Surg Laparosc Endosc Percutan Tech.

